# Fentanyl assisted treatment: a possible role in the opioid overdose epidemic?

**DOI:** 10.1186/s13011-019-0241-2

**Published:** 2019-11-11

**Authors:** Geoff Bardwell, Evan Wood, Rupinder Brar

**Affiliations:** 1 British Columbia Centre on Substance Use, 400-1045 Howe Street, Vancouver, BC V6Z 2A9 Canada; 2 Department of Medicine, University of British Columbia, 400-1045 Howe Street, Vancouver, BC V6Z 2A9 Canada; 3 Department of Family Practice, University of British Columbia, 5950 University Boulevard, Vancouver, BC V6T 1Z3 Canada

**Keywords:** North America, Opioid overdose epidemic, Opioid agonist treatment, Fentanyl assisted treatment

## Abstract

**Background:**

The current opioid overdose epidemic affecting communities across North America is increasingly driven by illicitly manufactured fentanyl and its related analogues. A variety of public health interventions have been implemented and scaled up, including opioid agonist treatments (OAT). While these treatments are successful for many individuals, they have a variety of limitations. It is critical to trial alternative treatments if conventional opioid agonist treatment options are not successful for a proportion of patients who use illicit fentanyl.

**Main body:**

Prescription fentanyl has been widely used for pain management. The use of transdermal fentanyl, specifically, which provides long acting and stable drug levels post-titration over several days, should be explored as an opioid agonist treatment option. The use of transdermal fentanyl for patients who use illicit fentanyl is currently being piloted in Vancouver, Canada. To address potential diversion, the patch is signed, dated, and a film dressing is applied to mitigate tampering. Evaluation outcomes are still pending, but there have been no adverse outcomes thus far and clinical improvements have been noted for many patients. This exploratory therapeutic approach should be considered across multiple settings and rigorously evaluated.

**Conclusions:**

There are known limitations to existing OAT options and there is a need to urgently evaluate alternative strategies for patients who are using illicit fentanyl not successfully treated with conventional OAT. Many patients may be attracted to, and retained in, fentanyl assisted treatment. This may be another strategy for certain patients to prevent harms caused by illicit fentanyl use, including overdose and death.

## Background

North America remains in the midst of an opioid overdose epidemic [1, 2]. While prescription opioid overdoses continue to drive overdose deaths in some settings, in many regions, the continued rise of overdose mortality is due to the proliferation of illicitly-manufactured fentanyl and its related analogues, which are significantly more potent than other illicit opioids (e.g., heroin) [3]. In the United States, fentanyl-related overdose deaths accounted for approximately 42% of all overdose deaths in 2017 [4]. In the Canadian province of British Columbia (BC), fentanyl and its analogues were detected in 87% of illicit drug toxicity deaths in 2018 [5]. Given the significant impact of illicit fentanyl attributable drug overdose deaths, a range of public health responses have been implemented in a variety of jurisdictions, including naloxone training and distribution, drug checking, and overdose prevention sites [6]. Despite the scale-up of these interventions, overdose death rates remain high, and novel response strategies are urgently needed to address this public health emergency.

There has been a longstanding need to expand access to opioid agonist treatment (OAT) to address the challenges of the opioid overdose crisis. However, while existing traditional OAT approaches (i.e., methadone and buprenorphine) are successful for many patients, these options have limitations such as limited ability to attract and retain patients in treatment [7]. In particular, these OAT options may have unwanted side effects for some patients [8] and often require daily presentation for witnessed ingestion at a methadone program or pharmacy. While existing OAT options do offer tremendous benefit, and are effective for many individuals, the limitations of these treatment options suggest a potential role for alternative approaches that may be better able to attract and retain patients in treatment in the fentanyl era. Additionally, current available evidence for the efficacy of OAT is in the context of illicit opioid use (e.g., heroin, prescription opioids) prior to the proliferation of fentanyl. Anecdotally, many patients are refractory to traditional OAT regarding sustained abstention from illicit fentanyl, though more research is needed in this area to fully characterize the effects of these OAT options. Recently, a study found fentanyl detected in the urine samples of more than 50% of participants that were enrolled in various OATs, demonstrating further limitations to existing treatments [9]. Moreover, while many individuals are ambivalent or have negative feelings about the proliferation of fentanyl in street-acquired drug supplies, emerging evidence also indicates that some individuals are now increasingly seeking fentanyl, not only because of its availability, but also due to its increased strength and the emergence of high tolerance to traditionally used street-acquired opioids [10]. In BC, for example, intentional use of illicit fentanyl has more than tripled in the last 3.5 years [11]. Thus, it is critical to trial alternative options if conventional OAT options are not successful for patients who use fentanyl.

## Main text

Following the logic of all OAT models whereby a long acting opioid is prescribed to treat illicit heroin use, we argue that the same type of approach should be explored for the treatment of illicit fentanyl use. Prescription fentanyl has been widely used for pain management. It can be administered orally, transdermally, and intravenously [12]. Additionally, fentanyl is inexpensive [12]. Fentanyl as an injectable is currently only used in anesthesia settings due to respiratory drive suppression, and thus presents pharmacologic risk outside of these settings. However, the use of transdermal fentanyl, which provides long acting and stable drug levels post-titration over several days, warrants further exploration as an innovative potential therapeutic approach.

The use of fentanyl patches for treatment of fentanyl use disorder is currently being piloted in Vancouver, BC, where there are eight patients (six males, two females; average age of 45) enrolled in the program. The pilot started in July 2019 and the selection criteria encompasses participants who use illicit fentanyl and have not benefited from oral OAT nor injectable OAT. All participants have a fixed address and are polysubstance users. Patients are started on a fentanyl patch titration, where the patch is changed every 2 days by a nurse. Diversion is important to consider as the patches can be tampered with and potentially sold on the street. In our setting, to address potential diversion, the patch is signed and dated and a transparent film dressing (i.e., Tegaderm) is applied to mitigate any tampering issues. Tampered or missing patches result in transitioning the patient to a different form of OAT. This pilot project is in its early stages, so evaluation outcomes are still pending. However, there have been no adverse outcomes thus far and clinical improvement has been noted for many patients.

Given the scope of the opioid overdose epidemic and the fact that it is largely driven by illicit fentanyl, implementation of this exploratory therapeutic approach coupled with rigorous evaluation should be an immediate priority. It could be implemented in various settings, including hospitals, inner city health and social services, and pharmacies. Evaluation efforts should focus on treatment uptake, adherence, and retention; suppression of illicit fentanyl use and other opioids; exposure to more lethal analogues (e.g., carfentanil) and other contaminants; impacts on quality of life; unintended effects; diversion; and patient linkages to other health and social services.

## Conclusions

In conclusion, a range of existing OAT options are effective for some patients, but not all. The increasing availability of illicit fentanyl is changing the treatment equation due to the need to move urgently to pursue strategies to better attract and retain high-risk individuals in OAT. In an era where existing OAT has known limitations for some, and where individuals are predominantly using fentanyl in many settings, some patients may be attracted to, and retained in, fentanyl assisted treatment programs. As long as diversion and safety are sufficiently addressed, fentanyl assisted treatment may be another strategy to preventing harms caused by illicit fentanyl use, including overdose and death.

## Data Availability

Not applicable.

## Abbreviations

BC: British Columbia
OAT: Opioid agonist treatment
